# 37. More than meets the eye: a 22 year old male with pes planus and hypermobility

**DOI:** 10.1093/rap/rky034

**Published:** 2018-09-20

**Authors:** Richard Smith, Sarah Bartram, Luke Sammut

**Affiliations:** 1 Rheumatology, Salisbury Foundation Trust, Salisbury, UNITED KINGDOM; 2 Rheumatology, Universtity Hospital Southampton Foundation Trust, Southampton, UNITED KINGDOM

**Introduction:** A 22 year old Caucasian male runner with a history of autism was referred to the lower limb clinic by the community podiatrist with painful flat feet which had not been helped by long term orthotics. The case report is a summary of events over a ten year period, highlighting the similarity of certain features, the potential for diagnostic confusion and the challenge in diagnosing rare inherited metabolic disorders.

**Case description:** A 22 year old Caucasian male runner with a history of autism was referred to the lower limb clinic by the community podiatrist with painful flat feet which had not been helped by long term orthotics. He was noted to be significantly taller than his mother and siblings at 195 cm with an arm span 194cm. Physical examination revealed joint hypermobility (Beighton score 9/9). A high arched palate and fish mouth scar were noted. The patient had a soft systolic murmur and reduced sensation to light touch in the stocking distribution bilaterally. His grandfather died young (late 40s) of a suspected myocardial infarction. A nerve conduction study confirmed peripheral neuropathy and a trans-thoracic echo cardiogram showed a possible patent ductus arteriosus only. A subsequent screen revealed B12 deficiency blood tests were negative for antibodies to Intrinsic factor and parietal cells. B12 was corrected with parenteral administration of B12 and the peripheral neuropathy improved. Further correspondence from the GP revealed that a maxillary facial surgeon had questioned Marfans as a child because of teeth overlap as had an optometrist because of eye problems. Subsequent referral to o phthalmology revealed bilateral phacodonosis (wobbly lens) and left ectopia lentis. Eye surgery was performed. The patient was later referred for genetic assessment as an inherited disorder of connective tissue was suspected. Marfan's screen (FBN1 gene) was negative but he was deemed by the geneticist to fulfil the Ghent criteria for Marfans and a presumed diagnosis of Marfans was made. Five years later the patient fractured his left tibia jumping from a groyne on a beach. This healed within thirteen weeks in a cast. Eight years after initial presentation the patient presented back to the lower limb clinic with neck pain, nausea and vertigo obe week post physiotherapy on his neck. Examination revealed no nystagmus but he did have an up going left plantar. The question was raised whether the patient had developed Bow-Hunter's syndrome (rotational vertigo). An urgent MRA revealed an occluded left internal carotid artery presumed due to dissection (not seen) and a left thalamus CVA. He was referred to the stroke unit and commenced on clopidrogrel and aspirin. An ultrasound doppler showed no obvious dissection. During admission, he developed a severe headache, a repeat MRI scan confirmed new onset left transverse sinus thrombosis. A thrombophilia screen revealed a homocysteine level of 362.5 umol/l (2 to 14.3 umol/l NR). An addition, blood tests also confirmed antithrombin III deficiency. A subsequent DXA scan confirmed osteopenia. Formal anticoagulation with rivaroxaban, an oral Factor Xa inhibitor, was commenced and the patient remains well with no neurological deficit. In summary, the patient was diagnosed with homocystinuria and antithrombin III deficiency. Pyridoxine was commenced but this had little effect on homocysteine levels. Further genetic screening was performed on patient and siblings.

**Discussion:** Analysis of CBS gene showed compound heterozygosity for both c919G. A substitution in exon 10 predicting a p.Gly307Ser, and a c.1136G>A substitution in exon 12, predicting a p.Arg379Gln missense variant. The former is the recurring Celtic variant associated with a pyridoxine non-responsive homocystinuria. It is likely both parents carry one of these gene variants. His sister had a slightly high homocysteine level and no action was required In this genetic variant, pyridoxine does not reduce homocysteine levels and might be toxic. In this situation, betaine (amethyl derivative of the amino acid glycine) may be prescribed. The diagnosis of Marfans had been queried by a surgeon and an optometrist. The patient had been referred with a simple biomechanical foot problem but examination revealed several features of Marfans, however strict interpretation of the revised 2010 Ghent criteria would not allow a definitive diagnosis. The 2010 Revised Ghent Nosology for Marfan syndrome relies on seven rules as indicated below: In the absence of family history: aortic root dilatation Z score ≥ 2 and ectopia lentis leads to Marfan syndrome. Aortic root dilatation Z score ≥ 2 and FBN1 leads to Marfan syndrome. Aortic root dilatation Z score ≥ 2 and systemic score ≥ 7pts leads to Marfan syndrome. Ectopialentis and FBN1 with known aortic root dilatation leads to Marfan syndrome. Absence of a mutation in the FBN1 gene should lead to investigation of other conditons presenting with Marfanoid habitus. It is unclear whether the Antithrombin III deficiency is related to homocysteinuria or is a unique diagnosis. The presence of antithrombin III deficiency is associated with a 60% increase risk in thrombosis (venous>arterial). This risk is increased in the presence of other hypercoagulable states. It is unclear form the literature whether diagnosis and management of homocystinuria as a child would have influenced the development of autism.

**Key Learning Points:** Homocystinuria is a recognised but frequently overlooked cause of vitamin B 12 (and B 2,6 and 9) deficiency. Marfans and homocystinuria have many overlapping features including a classic Marfanoid habitus and ectopialentis. Homocystinuira patients classically have Pes cavus not planus. A lack of mutation in the FBN1 gene should lead to investigation for other conditions (Shprintzen Goldberg syndrome, Loeys-Dietz syndrome, vascular Ehlers Danlos syndrome or homocystinruia). The life expectancy of patients with homocystinuria is reduced only if untreated. It is known that before the age of 30, almost one quarter of patients die as a result of thrombotic complications. Current NICE guidance for investigation of children with autism mention chromosome disorders as a medical differential but do not specifically mention homocystinuiria.

**Disclosure: R. Smith:** None. **S. Bartram:** None. **L. Sammut:** None.

